# 

**Published:** 1848-04

**Authors:** 


					AQEWTS OF TSZ8 JO
3. P. Laird, M. D. Columbus, 6a.
S. Parmly, M. D. New Orleans.
6. McDonald, M. D. Macon, Ga.
W. H. Goddard, Louisville, Ky.
James Taylor, M.D. Cincinnati, Ohio.
A. Baldwin, D. D. 8. Alabama.
John Lock, D. D. 8. Southport, Wis.
J?. H. Pebbles, Lexington, Mo.
J. C. Koss, Nashville, Tenn.
J. M. McCurdy, Philadelphia. Pa
M. Z. Kbeider, M. D. Lancaster, Ohio. 1
J. It. Penh,
Nicholas Cldte,
Benj. Strickland,
B. B. Brown, M.D. St.
w. R. Scott, P. D.
JS. M. Shepheed, Ml
John A, Cleveland, i
W. F. Bason, D. D. S. N.
John Lees, 23 Exchange Arcade, Ma
Chester, England. 4

				

